# Optimum Nitrogen and Phosphorus Combination Improved Yield and Nutrient Use Efficiency of Sorghum in Saline Soil

**DOI:** 10.3390/plants14010102

**Published:** 2025-01-02

**Authors:** Xiaoqian Guo, Qidi Wu, Luqi Wang, Guisheng Zhou, Guanglong Zhu, Mohamed Suliman Eltyb Suliman, Nimir Eltyb Ahmed Nimir

**Affiliations:** 1 College for Overseas Education, Yangzhou University, Yangzhou 225000, China; 2 China-Sudan Joint Laboratory of Crop Salinity and Drought Stress Physiology, The Ministry of Education of China, Yangzhou 225000, China; 3 Jiangsu Provincial Key Lab of Crop Genetics and Physiology, Yangzhou University, Yangzhou 225000, China; 4 Joint International Laboratory of Agriculture and Agri-Product Safety, Yangzhou University, Yangzhou 225000, China; 5 College of Agriculture, Nanjing Agricultural University, Nanjing 210095, China; 6 Faculty of Agriculture, University of Khartoum, Khartoum 11115, Sudan

**Keywords:** nitrogen, phosphorus, salinity, yield, nutrient use efficiency

## Abstract

Two-year experiments were conducted to assess the responses of yield and nutrient use efficiency of sorghum to nitrogen and phosphorus under saline soils. Three nitrogen rates (0, 180, and 360 N kg ha^−1^) and three phosphorus rates (0, 60, and 120 P_2_O_5_ kg ha^−1^) were used in this study. Our results showed that nitrogen and phosphorus supply increased SPAD (leaf greenness, 5.0–29.1%), NSC (non-structural carbohydrates, 14.0–41.2%), nutrient accumulation (nitrogen: 14.1–50.0%, phosphorus: 11.8–41.5%, potassium: 13.7–28.2%), biomass (fresh: 10.8–29.3%, dry: 5.0–22.8%), yield (16.6–30.7%), and harvest index (2.0–9.8%) of sorghum at the maturity stage grown in saline soils but significantly decreased nutrient use efficiency. Combined application showed better performance on these attributes than sole nitrogen and sole phosphorus. The highest seed yield (5919 kg ha^−1^) was recorded at 180 N kg ha^−1^ and 60 P_2_O_5_ kg ha^−1^ treatment, while the largest value of dry biomass (18,401 kg ha^−1^) was obtained at 360 N kg ha^−1^ and 120 P_2_O_5_ kg ha^−1^ treatment. The Pearson analysis showed that seed yield had the highest correlation with aerial potassium accumulation, while dry biomass had the highest correlation with NSC as well as aerial nitrogen accumulation. A treatment of 180 N kg ha^−1^ and 60 P_2_O_5_ kg ha^−1^ was recommended to be used in sorghum production in saline soils based on harvest index and nutrient use efficiency.

## 1. Introduction

Salt-affected soil (SAS) is a kind of soil that is chemically degraded under the constraints of high matrix and osmotic stress for crop plants due to the excessive soluble salts and exchangeable sodium [1]. Soil salinity threatens the growth and productivity of crop plants and hinders the sustainable development of global modern agriculture. It is estimated that at least one-third of the world’s cultivated land is affected by salinity, and this situation is expected to deteriorate further because of irrational land exploitation and global climate change [2]. Facing the challenge of feeding 9.3 billion people by 2050, there is an urgency for cultivating salt-tolerant crops and utilize salinized soil rationally [3].

Sorghum (*Sorghum bicolor* L. Moench) belongs to the gramineous family and ranks fifth in cereals after rice, wheat, barley, and maize based on its global production [4]. Due to its strong adaptability and moderate drought and salt tolerance [5,6], sorghum has the potential of being cultivated on marginal lands, such as barren slopes and saline–alkali lands and thus reduces competition with other food crops on fertile farmlands [7,8]. Fu et al. reported that about 1.95 × 10^4^ ha of marginal lands in northern China could be used for sorghum planting [9]. Nevertheless, sustained exposure to salt stress could affect its growth and thus productivity [10].

Under salinity stress, the impacts of salt ions on phytoavailability, absorption, transport, or distribution of nutrients in plants usually lead to nutrient imbalances, resulting in a remarkable decrease in crop yield and quality [11]. One of the vital strategies for dealing with the adverse effects of salt on plants is to adopt appropriate fertilization management practices [4]. Regarding saline soils, nitrogen (N) fertilization is the most effective approach to mitigate salt stress and maintain crop yield and quality [12]. Lin et al. claimed that N application enhanced chlorophyll content, hence increasing the photosynthesis and yield of oat in saline soil [13]. Zahedifar et al. reported that the negative effects of salinity on the yield of tomato were alleviated by N application [14]. Zamani et al. also found that applying N fertilizer significantly mitigated the damage of salt stress to sorghum by maintaining appropriate water potential, reducing ROS production and lipid peroxidation and enhancing the ratio of K^+^/Na^+^ [15]. Phosphorus (P) is the main nutrient that limits crop growth and development and alleviates salt-induced yield reduction [16]. Applying P fertilizer under saline conditions can improve crop yield. Sima et al. reported that the enhancing effect of P on salt tolerance in barley was related to the accumulation of mineral ions for osmoregulation and the limitation of Na^+^ accumulation in shoots [17]. Bouras et al. indicated that P fertilizer at 108 kg P_2_O_5_ ha^−1^ enhanced fresh biomass of blue panicum by 32% under 12 dS·m^−1^ [18]. Belouchrani et al. observed that P fertilization caused a significant enhancement of sorghum growth and salt tolerance, where it enhanced dry biomass, plant height, proline accumulation, and N and P uptake [19].

However, most studies have mainly focused on the effects of sole fertilizer on sorghum growth under salinity stress, with little research on the interactions of N and P. In addition, the improper application of P and N fertilizer not only decreases their efficiency but also results in environmental pollution [20]. Based on these, the objective of this study was to evaluate the alleviative effects of N and P fertilizer on sorghum yield and nutrient use efficiency in saline soils and to select the appropriate N rate as well as P rate for practical use.

## 2. Materials and Methods

### 2.1. Experimental Cultivar and Site

The experiment was conducted in Dafeng Coastal Forest Farm, Yancheng City, China (33°20′ N, 120°47′ E) in the sorghum growing seasons of 2021 and 2023 (due to the COVID-19, we did not conduct the field experiment in 2022). The tested sorghum cultivar was ‘Jitian 3’, which was provided by Hebei Academy of Agriculture and Forestry Sciences. The cultivated layer of experimental land contained 19.75 g kg^−1^ organic matter, 279 mg kg^−1^ available potassium, 1.45 mg kg^−1^ available P, and 0.72 g kg^−1^ total N, and the soil pH reading and electrical conductivity were 8.8 and 10.87 mS cm^−1^, respectively.

### 2.2. Experimental Design

The experiment was a two-factor randomized block design with three replications. Three N fertilizer levels (0, 180, and 360 N kg ha^−1^, denoted by N0, N1, and N2) and three P fertilizer levels (0, 60, and 120 P_2_O_5_ kg ha^−1^, denoted by P0, P1, and P2, respectively) were used in this study. There were 27 plots in total, each with an area of 30 m^2^ (15 m × 2 m). The method of burrowing was adopted, with 3 seeds in each hole. The plant spacing was 15 cm × 50 cm. Phosphate fertilizer was applied in the form of superphosphate as the base fertilizer, and N fertilizer was applied in the form of urea with a ratio of base fertilizer:jointing fertilizer:booting fertilizer = 4:3:3. Other field managements were performed according to local recommendations.

### 2.3. Sampling and Measurement

At the seedling, jointing, and maturity stages, leaf chlorophyll content was assessed by using a portable chlorophyll meter (SPAD-502, Minolta Corporation, Tokyo, Japan). Five sorghum plants were randomly selected from each plot, and SPAD readings were average values which were obtained from the top, middle, and bottom portion of the third top leaf.

At the three sampling stages mentioned above, five sorghum plants were randomly selected from each plot. After weighing the fresh weight, plant samples were put into an oven and dried at 105 °C for 30 min to deactivate enzymes and subsequently dried at 80 °C to a constant weight for the measurement of dry weight. All the samples were ground to determine the content of soluble sugar and starch and the concentration of N, P, and K. Soluble sugar and starch content were determined as described by Gao [21]. N concentration was measured according to the micro-Kjeldahl method [22]. P and K concentrations were measured followed the method of Hao [23]. The non-structural carbohydrate (NSC) content was calculated as follows:

$$NSC\left(mg\;g^{-1}\right)=Soluble\;sugar+Starch$$

Ten representative sorghum plants were harvested from each plot at the maturity stage. The number of grains per plant, grain weight per plant, 1000-grain weight, and yield were determined. The harvest index (HI) was calculated as follows:

HI=Seed yield/(Seed yield+Aerial plant dry weight)

N accumulation is the product of plant N concentration and dry weight. The N use efficiency (NUE) was calculated as follows:

$$NUE\left(kg\;{kg}^{-1}\right)=Seed\;yield/N\;accumulation$$

P accumulation is the product of plant P concentration and dry weight. The P use efficiency (PUE) was calculated as follows:

$$PUE\left(kg\;{kg}^{-1}\right)=Seed\;yield/P\;accumulation$$

K accumulation is the product of plant K concentration and dry weight. The K use efficiency (KUE) was calculated as follows:

$$KUE\left(kg\;{kg}^{-1}\right)=Seed\;yield/K\;accumulation$$

### 2.4. Statistical Analysis

Microsoft Excel 2016 was used to input and calculate the test data. SPSS 22.0 was used for an analysis of variance, and the Duncan 0.05 method was used for multiple comparisons according to the design of the experiment. A Pearson correlation analysis was performed and graphs were plotted with Origin 2024. All parameters are presented as the average of the 2-year experiment, as the trends of each parameter are similar in each year. Data of 2-year experiments have been attached as Supplementary Materials.

## 3. Results

N and P exhibited significant influences on aerial fresh weight and dry weight (Table 1 and Table S1). Compared with applying N or P only, the aerial fresh and dry weight were higher under the combined application of N and P. At the N0 and N1 levels, aerial fresh and dry weight with P2 treatment were lower than those with P1 treatment, while the reverse trends were observed at the N2 level. At the three sampling stages, the largest increase percentages of aerial plant fresh weight and dry weight were obtained in the N2P2 and N1P1 treatments (29.3% and 26.4% for fresh weight and 22.8% and 20.6% for dry weight, respectively, at the maturity stage).

N and P significantly affected seed yield and yield components of sorghum under saline soils except 1000-seed weight (Table 2 and Table S2). N and P enhanced seed weight per spike, seed number per spike, and yield, but the highest level (N2 and P2) led to slight decreases in these attributes. Compared with N only (N1P0 and N2P0), the application of P (N0P1 and N0P2) had better improvement on seed weight per spike, seed number per spike, and yield. Seed weight per spike, seed number per spike, and yield had the maximum values at N1P1, increasing, respectively, by 30.6%, 40.3%, and 30.7% as compared with N0P0, followed by N1P2 and N2P2.

**Figure 1:**
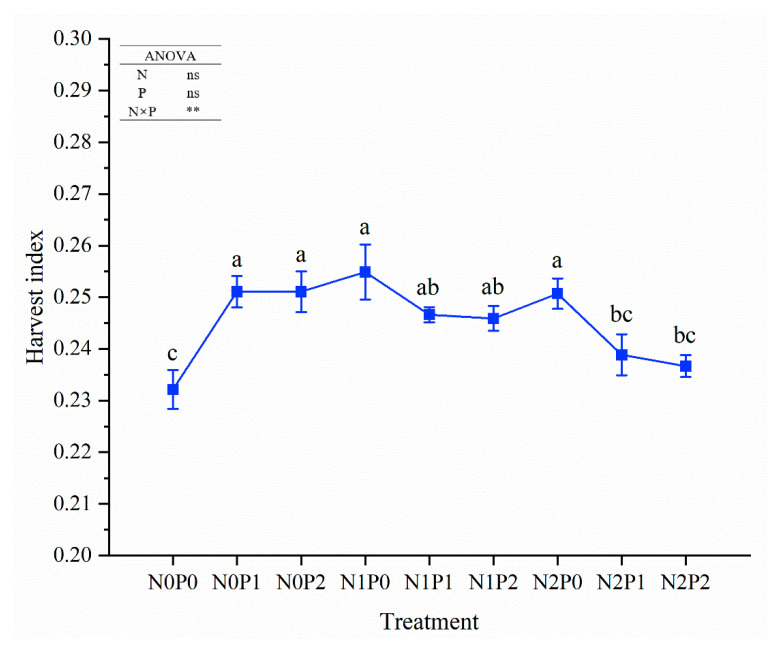
Effects of nitrogen and phosphorus on harvest index of sorghum grown in saline soils. ns: non-significant difference; **: significant difference at *p* ≤ 0.01. Different lowercase letters indicate significant level of difference among different treatments at *p* ≤ 0.05.

The interaction effects of N and P on harvest index were significant, while single N application or P application had no significant effects (Figure 1 and Figure S1). N1P0 increased harvest index to the highest level (increased by 9.8%). The harvest indexes under the combined application of N and P were all lower than those from treatment with individual application of N or P. Although N2P2 produced the lowest harvest index, its harvest index was still higher than that of the control.

N and P had significant effects on SPAD readings and NSC content of sorghum grown in saline soils (Table 3 and Table S3). Compared with N1P0 and N2P0, sorghum plants treated with N0P1 and N0P2 had higher NSC content, while SPAD readings showed the reverse trend. In general, however, the best improvements in SPAD readings and NSC content were found under the combined application of N and P. Among all the treatments, N2P2 produced the highest SPAD readings and NSC content, with an increase of 29.3% and 39.6% at the seedling stage, 26.5% and 37.4% at the jointing stage, and 29.0% and 41.2% at the maturity stage, respectively.

N accumulation in the aerial part of sorghum grown in saline soils increased with the application of N and P (Table 4 and Table S4). Compared with applying N or P only, the aerial N accumulation was higher under the combined application of N and P. Among these four combined treatments, N2P2 produced the largest increase percentage in N accumulation, while N1P2 had the smallest increase. NUE had the reverse trend. At the maturity stage, N2P2 produced the highest value of aerial N accumulation (increased by 50.0%) but decreased NUE by 16.0%.

Aerial P accumulation had similar trends with N accumulation (Table 5 and Table S5). The application of N and P remarkably enhanced the aerial P accumulation. The highest aerial P accumulation among the four combined treatments was also recorded at N2P2 (increased by 41.5% at the maturity stage), but the worst performance was observed at N2P1. At the N0 level, P had no significant effects on PUE, but at the N1 and N2 levels, the application of P remarkably decreased PUE. The lowest PUE was recorded at N1P2 and N2P2 (decreased by 9.9% and 11.0%, respectively).

The application of N and P promoted the uptake of K by sorghum grown in saline soils but had no significant effects on the KUE (Table 6 and Table S6). The largest aerial K accumulation at three sampling stages were all obtained at N1P1 (increasing by 31.5% at the seedling stage, 26.7% at the jointing stage, and 28.2% at the maturity stage, respectively). Interestingly, N0P1 also increased aerial K accumulation to the same level with the combined application of N and P.

**Figure 2:**
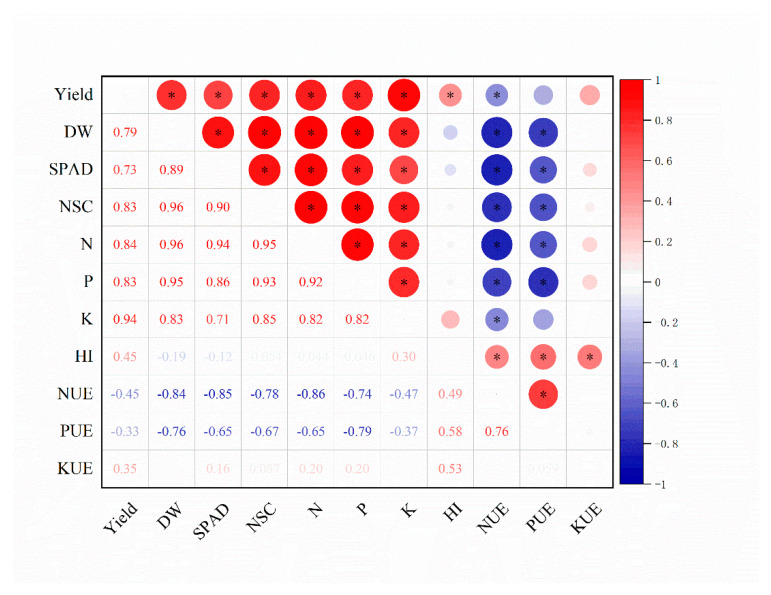
Relationships between all measured characteristics of sorghum grown in saline soils. DW: dry weight; N: aerial N accumulation; P: aerial P accumulation; K: aerial K accumulation. *: significant difference at *p* ≤ 0.05.

The Pearson analysis showed that both yield and dry weight had positive correlations with other physiological parameters of sorghum grown in the saline soils (Figure 2 and Figure S2). The correlation coefficient was the largest between seed yield and aerial K accumulation, followed by N accumulation. Yield also had positive correlations with harvest index and KUE, respectively, whereas the negative correlations were observed between yield and NUE and between yield and PUE. The largest correlation coefficients were found between dry weight and NSC as well as aerial N accumulation, while the smallest was obtained between dry weight and K accumulation. Different from the situation of yield, aerial dry weight had negative correlations with harvest index and nutrient use efficiency.

## 4. Discussion

The rational input of these two nutrients could advance the accumulation of biomass, which is fundamental to the formation of high yield [24,25]. In our study, seed yield and yield components of sorghum were increased with the application of N and P in saline soils. Similar results were reported by Bouras et al. [26] and Ma et al. [27]. However, N and P had no significant effects on 1000-seed weight, indicating that the increase in seed yield was mainly the result of the enhancement of seed number per spike. The supply of fertilizer provided sufficient nutrients for young spike differentiation and reduced spikelet degradation, so as to promote seed number per spike. Moreover, our study showed that the highest seed yield was recorded at N1P1 rather than the largest fertilizer rate (N2P2). Thus, a heavy application of fertilizer is not always beneficial.

In our study, sorghum biomass increased with the application of N and P. This increase in biomass could be attributed to the fact that N is an essential component of chlorophyll, amino acids, protein, and enzymes and plays a vital role in cell formation and cell structure [28]. On the other hand, P is an important macronutrient that promotes root growth and the absorption of soil nutrients, which might also be the main cause for enhancing biomass. However, different from yield, the greatest sorghum biomass was obtained at N2P2. Combined with yield, those results verified that excess fertilizer might decrease seed yield by advancing excessive vegetative growth, resulting in the increased possibility of crop lodging and delaying crop maturity in non-saline and saline soils [29]. In addition, our results also showed that the combined supply of N and P had better performance on improving biomass and yield of sorghum under saline soils compared with sole N and sole P, whereas a reverse trend was observed on harvest index. The greatest application rate, N2P2, produced the lowest harvest index, but it was still higher than that of the control. Thus, the application rate of fertilizer should be consistent with the demand of crops across the entire growth period and the objectives of production, especially under salinity stress.

The reduction in yield under salinity is mainly explained by decreased photosynthetic activity [30]. In this study, SPAD readings increased when sorghum plants were treated with N and P in saline soils, which attributed to increased chlorophyll content. In addition, this study showed that SPAD readings of the plants treated with N were more increased than those treated with P. N is the basic element for chlorophyll synthesis and plays a crucial role in crop photosynthesis [31,32]. The enhancement in chlorophyll content with N could increase amino acid, protein and enzymes, which play an important role in photosynthetic production and yield formation [28].

Carbohydrates in plants consist of structural and non-structural types. Structural types are polysaccharides, which are the components of cell wall and provide structural support for plants, while non-structural carbohydrates (NSCs) act as intermediate metabolism as well as energy transport and storage in plants [33,34]. Similar to SPAD, in our study, NSC content enhanced with the addition of N and P, and the highest values were all observed at N2P2 and N1P1. The utilization of fertilizers increased photosynthetic capacity of plants, providing more photosynthates for crop growth, which was confirmed by sorghum biomass in this study. However, our results did not agree with a previous study where both N and P fertilizer reduced the concentrations of NSC of aquatic fern (*Salvinia minima* Baker) [35]. This might be due to the differences in species and growth environment. At the jointing stage, NSC content had lower values than the other two stages, suggesting that most of the NSCs were used to constitute the plant structures.

Achieving significant yield depends partly on identifying the nutritional requirements of crops throughout the growth cycle and the specificities of the environment in which such cultivation involves [19]. This study showed that N and P application significantly increased the accumulation of N, P, and K of sorghum plants, indicating that the application of N and P also promoted the absorption of other nutrients by crops. In our study, the biomass and seed yield of sorghum were positively and significantly related with the accumulation of all the three macronutrients of N, P, and K. This verified that fertilizer application could increase yield by improving the absorption of nutrient by crops. By comparing the positive effects of fertilizer supply on nutrient accumulation, we found that N accumulation had the most increments among the three macroelements. Previous studies also exhibited that N accounts for around 80% of the total mineral nutrients absorbed by plants in most soils, and it is normally the most growth-limiting nutrient for plants [36,37], which supports the results of our study. Moreover, except for increasing nutrient absorption, reasonable fertilizer application also reduced Na^+^ accumulation in plants [38]. Therefore, we suggest that appropriate N and P application played not only a nutritional role but also a vital role in improving salt tolerance by increasing the absorption of other nutrients and reducing Na^+^ accumulation in plants.

However, in our study, the utilization of fertilizer decreased nutrient use efficiency, including NUE and PUE, and the negative effects was more pronounced with an increased fertilizer rate. Nutrient use efficiency is determined by seed yield and nutrient accumulation in whole plants. Our results suggested that although seed yield was increased with increased fertilizer rate, more nutrients were accumulated in the plant instead of being converted into yield. There is still room for improving nutrient use efficiency of sorghum under saline soils.

## 5. Conclusions

The application of N and P increased biomass, yield, and harvest index of sorghum grown in saline soils, but significantly decreased nutrient use efficiency. Aerial N accumulation contributed more to sorghum biomass, while the enhancement of seed yield was mainly caused by aerial K uptake. Considering harvest index and nutrient use efficiency, N1P1 (180 N kg ha^−1^ and 60 P_2_O_5_ kg ha^−1^) was recommended to be an appropriate fertilizer treatment to increase sorghum yield in saline soils.

## Figures and Tables

**Table 1:** Effects of nitrogen and phosphorus on fresh and dry biomass of sorghum at three growing stages grown in saline soils.

Nitrogen	Phosphorus	Aerial Fresh Weight (kg ha^−1^)			Aerial Dry Weight (kg ha^−1^)		
		Seedling	Jointing	Maturity	Seedling	Jointing	Maturity
N0	P0	744.0 ± 7.3 ^e^	4488 ± 96 ^f^	40,372 ± 530 ^f^	169.7 ± 2.5 ^f^	945 ± 10 ^g^	14,990 ± 159 ^g^
	P1	848.4 ± 3.4 ^c^	5160 ± 26 ^d^	47,409 ± 902 ^d^	190.9 ± 2.7 ^cd^	1057 ± 7 ^d^	16,638 ± 111 ^d^
	P2	847.4 ± 6.4 ^c^	5001 ± 58 ^de^	44,740 ± 259 ^e^	179.1 ± 1.4 ^e^	982 ± 13 ^f^	15,745 ± 96 ^f^
N1	P0	829.2 ± 10.3 ^cd^	5132 ± 43 ^de^	44,974 ± 220 ^e^	184.4 ± 1.9 ^de^	1021 ± 7 ^e^	15,884 ± 211 ^ef^
	P1	961.4 ± 12.8 ^a^	5523 ± 49 ^b^	51,045 ± 374 ^ab^	212.8 ± 4.1 ^a^	1143 ± 12 ^b^	18,082 ± 21 ^ab^
	P2	902.4 ± 12.9 ^b^	5327 ± 32 ^c^	48,477 ± 641 ^cd^	198.7 ± 2.6 ^bc^	1101 ± 3 ^c^	17,505 ± 111 ^c^
N2	P0	810.6 ± 8.4 ^d^	4988 ± 29 ^e^	45,005 ± 1009 ^e^	181.2 ± 1.8 ^e^	1030 ± 3 ^e^	16,266 ± 145 ^de^
	P1	924.1 ± 9.5 ^b^	5534 ± 74 ^b^	49,680 ± 611 ^bc^	203.7 ± 2.1 ^b^	1142 ± 13 ^b^	17,889 ± 174 ^bc^
	P2	979.3 ± 6.0 ^a^	5814 ± 24 ^a^	52,203 ± 578 ^a^	215.3 ± 4.2 ^a^	1176 ± 2 ^a^	18,401 ± 157 ^a^

Within each sampling date, the data followed with different letters are statistically different at the 0.05 probability level.

**Table 2:** Effects of nitrogen and phosphorus on yield and yield components of sorghum grown in saline soils.

Nitrogen	Phosphorus	Seed Weight per Spike (g)	1000-Seed Weight (g)	Seed Number per Spike	Seed Yield (kg ha^−1^)
N0	P0	46.9 ± 0.5 ^d^	25.6 ± 0.9 ^ab^	1838 ± 68 ^d^	4530 ± 47 ^d^
	P1	57.7 ± 0.7 ^bc^	25.4 ± 0.4 ^ab^	2276 ± 45 ^bc^	5575 ± 68 ^bc^
	P2	54.6 ± 1.5 ^c^	23.8 ± 0.8 ^b^	2300 ± 20 ^bc^	5280 ± 143 ^c^
N1	P0	56.2 ± 1.3 ^bc^	26.0 ± 0.3 ^a^	2160 ± 45 ^c^	5432 ± 124 ^bc^
	P1	61.2 ± 0.5 ^a^	23.8 ± 0.8 ^b^	2578 ± 66 ^a^	5919 ± 49 ^a^
	P2	59.1 ± 1.1 ^ab^	24.9 ± 0.8 ^ab^	2382 ± 66 ^b^	5711 ± 111 ^ab^
N2	P0	56.3 ± 1.2 ^bc^	25.5 ± 0.4 ^ab^	2217 ± 11 ^c^	5443 ± 114 ^bc^
	P1	58.1 ± 0.8 ^b^	26.0 ± 0.3 ^a^	2236 ± 33 ^bc^	5612 ± 75 ^b^
	P2	59.0 ± 0.2 ^ab^	26.8 ± 0.3 ^a^	2211 ± 32 ^c^	5705 ± 18 ^ab^

Within each column, the data followed with different letters are statistically different at the 0.05 probability level.

**Table 3:** Effects of nitrogen and phosphorus on SPAD reading and NSC of sorghum grown in saline soils at three growing stages.

Nitrogen	Phosphorus	SPAD Reading			Aerial NSC Content (mg g^−1^)		
		Seedling	Jointing	Maturity	Seedling	Jointing	Maturity
N0	P0	30.2 ± 0.4 ^g^	37.2 ± 0.5 ^e^	28.2 ± 0.4 ^g^	165.6 ± 0.8 ^h^	77.5 ± 0.4 ^e^	265.0 ± 5.8 ^f^
	P1	32.5 ± 0.4 ^ef^	41.4 ± 0.5 ^c^	30.4 ± 0.3 ^ef^	195.9 ± 0.8 ^e^	86.7 ± 0.3 ^d^	325.4 ± 4.7 ^c^
	P2	31.8 ± 0.4 ^f^	38.7 ± 0.7 ^d^	29.6 ± 0.6 ^fg^	200.9 ± 1.4 ^d^	93.1 ± 0.7 ^c^	308.3 ± 2.6 ^de^
N1	P0	33.5 ± 0.4 ^de^	39.7 ± 0.3 ^d^	30.5 ± 0.7 ^ef^	184.9 ± 1.6 ^g^	87.7 ± 1.0 ^d^	302.1 ± 3.3 ^e^
	P1	37.2 ± 0.1 ^bc^	45.6 ± 0.3 ^a^	34.3 ± 0.4 ^b^	226.6 ± 0.8 ^a^	103.9 ± 0.5 ^a^	366.4 ± 3.4 ^a^
	P2	36.0 ± 0.4 ^c^	43.2 ± 0.6 ^b^	32.4 ± 0.6 ^cd^	211.1 ± 1.8 ^c^	98.0 ± 1.3 ^b^	344.5 ± 2.5 ^b^
N2	P0	34.3 ± 0.2 ^d^	42.3 ± 0.1 ^bc^	31.4 ± 0.3 ^de^	190.0 ± 2.0 ^f^	87.7 ± 1.5 ^d^	317.9 ± 3.8 ^cd^
	P1	38.0 ± 0.6 ^b^	46.1 ± 0.6 ^a^	33.1 ± 0.3 ^bc^	220.6 ± 2.6 ^b^	98.8 ± 0.4 ^b^	348.2 ± 3.7 ^b^
	P2	39.1 ± 0.3 ^a^	47.1 ± 0.4 ^a^	36.4 ± 0.4 ^a^	231.1 ± 2.2 ^a^	106.5 ± 1.2 ^a^	374.3 ± 4.0 ^a^

Within each sampling date, the data followed with different letters are statistically different at the 0.05 probability level.

**Table 4:** Effects of nitrogen and phosphorus on aerial N accumulation and NUE of sorghum grown in saline soils at three growing stages.

Nitrogen	Phosphorus	Aerial N Accumulation (kg ha^−1^)			NUE (kg kg^−1^)
		Seedling	Jointing	Maturity	
N0	P0	1.932 ± 0.050 ^f^	11.38 ± 0.16 ^g^	123.3 ± 0.2 ^g^	36.8 ± 0.4 ^b^
	P1	2.234 ± 0.025 ^de^	13.27 ± 0.11 ^e^	146.8 ± 0.6 ^e^	38.0 ± 0.3 ^a^
	P2	2.133 ± 0.018 ^e^	12.23 ± 0.30 ^f^	140.7 ± 3.3 ^f^	37.5 ± 0.3 ^ab^
N1	P0	2.292 ± 0.011 ^cd^	13.61 ± 0.02 ^de^	148.5 ± 0.7 ^e^	36.6 ± 0.7 ^b^
	P1	2.966 ± 0.068 ^a^	16.63 ± 0.13 ^b^	180.0 ± 0.6 ^ab^	32.9 ± 0.4 ^d^
	P2	2.662 ± 0.032 ^b^	15.47 ± 0.23 ^c^	166.8 ± 3.4 ^c^	34.3 ± 0.1 ^c^
N2	P0	2.374 ± 0.025 ^c^	14.00 ± 0.13 ^d^	154.8 ± 1.7 ^d^	35.2 ± 0.4 ^c^
	P1	2.995 ± 0.048 ^a^	16.68 ± 0.20 ^b^	176.0 ± 1.2 ^b^	31.9 ± 0.3 ^de^
	P2	3.085 ± 0.057 ^a^	17.96 ± 0.08 ^a^	184.9 ± 1.7 ^a^	30.9 ± 0.4 ^e^

NUE: nitrogen use efficiency. Within each sampling date, the data followed with different letters are statistically different at the 0.05 probability level..

**Table 5:** Effects of nitrogen and phosphorus on aerial P accumulation and PUE of sorghum grown in saline soils at three growing stages.

Nitrogen	Phosphorus	Aerial P Accumulation (kg ha^−1^)			PUE (kg kg^−1^)
		Seedling	Jointing	Maturity	
N0	P0	0.301 ± 0.001 ^f^	1.923 ± 0.036 ^e^	32.7 ± 0.1 ^e^	138.8 ± 1.4 ^b^
	P1	0.376 ± 0.004 ^c^	2.259 ± 0.034 ^c^	39.4 ± 0.2 ^c^	142.0 ± 2.1 ^b^
	P2	0.356 ± 0.005 ^d^	2.130 ± 0.027 ^d^	37.9 ± 0.9 ^cd^	139.9 ± 0.8 ^b^
N1	P0	0.338 ± 0.002 ^de^	2.108 ± 0.004 ^d^	36.6 ± 0.7 ^d^	148.9 ± 0.6 ^a^
	P1	0.470 ± 0.011 ^a^	2.652 ± 0.030 ^b^	45.0 ± 0.2 ^ab^	131.9 ± 0.8 ^c^
	P2	0.463 ± 0.003 ^a^	2.618 ± 0.021 ^b^	45.8 ± 0.4 ^a^	125.1 ± 1.4 ^d^
N2	P0	0.331 ± 0.003 ^e^	2.120 ± 0.021 ^d^	37.2 ± 0.8 ^d^	146.7 ± 1.9 ^a^
	P1	0.436 ± 0.006 ^b^	2.593 ± 0.044 ^b^	43.6 ± 0.3 ^b^	129.2 ± 1.7 ^c^
	P2	0.481 ± 0.011 ^a^	2.779 ± 0.017 ^a^	46.3 ± 0.1 ^a^	123.6 ± 0.5 ^d^

PUE: phosphorus use efficiency. Within each sampling date, the data followed with different letters are statistically different at the 0.05 probability level.

**Table 6:** Effects of nitrogen and phosphorus on aerial K accumulation and KUE of sorghum grown in saline soils at three growing stages.

Nitrogen	Phosphorus	Aerial K Accumulation (kg ha^−1^)			KUE (kg kg^−1^)
		Seedling	Jointing	Maturity	
N0	P0	1.585 ± 0.023 ^d^	7.58 ± 0.05 ^e^	100.4 ± 0.2 ^e^	45.2 ± 0.4 ^a^
	P1	2.047 ± 0.027 ^a^	9.52 ± 0.07 ^a^	125.6 ± 0.9 ^ab^	44.5 ± 0.4 ^a^
	P2	1.780 ± 0.026 ^c^	8.39 ± 0.18 ^d^	114.2 ± 0.5 ^d^	46.3 ± 1.3 ^a^
N1	P0	1.910 ± 0.049 ^b^	9.04 ± 0.16 ^bc^	118.2 ± 1.7 ^c^	46.1 ± 0.6 ^a^
	P1	2.083 ± 0.054 ^a^	9.61 ± 0.22 ^a^	128.8 ± 0.9 ^a^	46.1 ± 0.7 ^a^
	P2	1.922 ± 0.020 ^b^	9.31 ± 0.08 ^ab^	123.5 ± 1.1 ^b^	46.3 ± 0.7 ^a^
N2	P0	1.885 ± 0.024 ^bc^	8.74 ± 0.04 ^cd^	119.4 ± 1.8 ^c^	45.7 ± 0.5 ^a^
	P1	2.048 ± 0.026 ^a^	9.48 ± 0.09 ^a^	123.5 ± 1.0 ^b^	45.5 ± 0.9 ^a^
	P2	2.051 ± 0.064 ^a^	9.60 ± 0.07 ^a^	125.4 ± 1.2 ^ab^	45.6 ± 0.5 ^a^

KUE: potassium use efficiency. Within each sampling date, the data followed with different letters are statistically different at the 0.05 probability level.

## Data Availability

Data are contained within the article and supplementary materials.

## Supplementary Materials

The following supporting information can be downloaded at https://www.mdpi.com/article/10.3390/plants14010102/s1, Table S1. Effects of nitrogen and phosphorus on fresh and dry biomass of sorghum at three growing stages grown in saline soils in 2021 and 2023; Table S2. Effects of nitrogen and phosphorus on yield and yield components of sorghum grown in saline soils in 2021 and 2023; Table S3. Effects of nitrogen and phosphorus on SPAD reading and NSC of sorghum at three growing stages grown in saline soils in 2021 and 2023; Table S4. Effects of nitrogen and phosphorus on aerial N accumulation and NUE of sorghum at three growing stages grown in saline soils in 2021 and 2023; Table S5. Effects of nitrogen and phosphorus on aerial P accumulation and PUE of sorghum at three growing stages grown in saline soils in 2021 and 2023; Table S6. Effects of nitrogen and phosphorus on aerial K accumulation and KUE of sorghum at three growing stages grown in saline soils in 2021 and 2023; Figure S1. Effects of nitrogen and phosphorus on harvest index of sorghum grown in saline soils in 2021 and 2023. (A) 2021; (B) 2023; ns: non-significant difference; **: significant difference at *p* ≤ 0.01; Figure S2. Relationships between all the measured characteristics of sorghum grown in saline soils in 2021 and 2023. (A) 2021; (B) 2023; DW: Dry weight; N: Aerial N accumulation; P: Aerial P accumulation; K: Aerial K accumulation. *: significant difference at *p* ≤ 0.05.
