# Supplementary material for: Optimum Nitrogen and Phosphorus Combination Improved Yield and Nutrient Use Efficiency of Sorghum in Saline Soil
Source: Plants (Basel). 2025 Jan 2;14(1):102. doi: 10.3390/plants14010102 (PMC11722898; doi:10.3390/plants14010102)
Supplement: Supplementary file 1 [file plants-14-00102-s001.zip › plants-3111374-supplementary.pdf]

Table S1. Effects of nitrogen and phosphorus on fresh and dry biomass of sorghum at three growing stages grown in saline soils in 2021 and 2023

Nitrogen	Phosphorus	Aerial fresh weight (kg ha^{-1})			Aerial dry weight (kg ha^{-1})		
		Seedling	Jointing	Maturity	Seedling	Jointing	Maturity
**2021**							
**N0**	**P0**	766.8±15.8^{e}	4620±101^{e}	40993±1095^{d}	171.3±2.4^{e}	956±18^{e}	15081±58^{e}
	**P1**	879.6±13.1^{d}	5360±27^{cd}	48303±1495^{bc}	193.8±3.3^{cd}	1072±23^{c}	16828±313^{c}
	**P2**	876.1±11.7^{d}	5165±107^{d}	45631±479^{c}	181.9±1.4^{de}	995±11^{d}	15861±219^{d}
**N1**	**P0**	859.6±9.3^{d}	5288±81^{cd}	45930±176^{c}	187.1±5.8^{d}	1037±10^{c}	16093±292^{d}
	**P1**	991.7±16.5^{ab}	5737±71^{b}	52026±435^{a}	215.5±3.5^{a}	1161±3^{a}	18246±163^{ab}
	**P2**	937.2±6.6^{c}	5508±54^{bc}	49255±312^{b}	201.5±4.6^{bc}	1117±5^{b}	17711±226^{b}
**N2**	**P0**	844.8±14.8^{d}	5159±41^{d}	45998±1420^{c}	184.3±4.4^{d}	1052±10^{c}	16460±112^{cd}
	**P1**	964.7±11.5^{bc}	5727±106^{b}	50642±413^{ab}	206.1±2.9^{ab}	1158±16^{a}	18060±33^{ab}
	**P2**	1019.0±7.4^{a}	6006±68^{a}	53120±772^{a}	218.1±4.6^{a}	1193±7^{a}	18535±138^{a}
**2023**							
**N0**	**P0**	721.1±12.2^{e}	4357±133^{e}	39751±36^{f}	168.0±3.9^{e}	935±14^{e}	14898±264^{e}
	**P1**	817.2±8.0^{c}	4960±50^{cd}	46515±309^{d}	188.1±2.2^{cd}	1042±9^{c}	16448±102^{c}
	**P2**	818.8±5.4^{c}	4837±24^{d}	43850±974^{e}	176.4±1.5^{de}	969±24^{de}	15629±148^{d}
**N1**	**P0**	798.9±11.5^{cd}	4976±99^{cd}	44018±534^{e}	181.6±2.7^{d}	1004±10^{cd}	15674±131^{d}
	**P1**	931.1±15.9^{a}	5310±59^{b}	50064±492^{ab}	210.1±4.8^{a}	1125±22^{a}	17918±124^{a}
	**P2**	867.6±19.3^{b}	5146±10^{bc}	47698±1302^{cd}	195.9±7.7^{bc}	1084±2^{b}	17300±47^{b}
**N2**	**P0**	776.3±6.2^{d}	4817±97^{d}	44012±601^{e}	178.1±0.9^{de}	1009±3^{cd}	16072±235^{cd}
	**P1**	883.5±17.2^{b}	5342±49^{b}	48718±809^{bc}	201.3±2.6^{ab}	1126±11^{a}	17718±314^{ab}
	**P2**	939.6±8.4^{a}	5623±24^{a}	51285±418^{a}	212.5±4.1^{a}	1158±9^{a}	18268±228^{a}

Within each sampling date, the data followed with different letters are statistically different at the 0.05 probability level.

Table S2. Effects of nitrogen and phosphorus on yield and yield components of sorghum grown in saline soils in 2021 and 2023

Nitrogen	Phosphorus	Seed weight per spike (g)	1000-Seed weight (g)	Seed number per spike	Seed yield (kg ha^{-1})
**2021**					
**N0**	**P0**	47.0±0.4^{e}	25.9±1.4^{ab}	1824±87^{c}	4543±35^{e}
	**P1**	57.6±0.9^{bcd}	25.2±0.7^{ab}	2291±39^{b}	5567±90^{bcd}
	**P2**	55.3±1.3^{d}	24.0±1.2^{b}	2308±65^{b}	5342±128^{d}
**N1**	**P0**	56.0±1.0^{cd}	26.1±0.9^{ab}	2146±78^{b}	5411±94^{cd}
	**P1**	61.7±0.7^{a}	23.9±1.6^{b}	2601±147^{a}	5968±65^{a}
	**P2**	60.0±1.9^{ab}	25.1±1.6^{ab}	2406±112^{ab}	5796±181^{ab}
**N2**	**P0**	56.3±1.4^{cd}	25.5±1.2^{ab}	2215±74^{b}	5439±136^{cd}
	**P1**	58.3±1.0^{abcd}	26.4±0.8^{ab}	2216±88^{b}	5640±97^{abcd}
	**P2**	59.5±0.6^{abc}	27.9±0.2^{a}	2136±34^{b}	5755±60^{abc}
**2023**					
**N0**	**P0**	46.7±0.7^{d}	25.3±0.6^{abc}	1851±72^{c}	4517±71^{d}
	**P1**	57.8±0.6^{ab}	25.6±0.9^{ab}	2260±93^{b}	5584±62^{ab}
	**P2**	54.0±1.7^{c}	23.6±0.6^{c}	2291±25^{b}	5219±165^{c}
**N1**	**P0**	56.4±1.6^{bc}	25.9±0.4^{a}	2174±48^{b}	5452±155^{bc}
	**P1**	60.7±0.3^{a}	23.8±0.1^{bc}	2555±16^{a}	5871±34^{a}
	**P2**	58.2±0.5^{ab}	24.7±0.5^{abc}	2357±62^{b}	5625±44^{ab}
**N2**	**P0**	56.3±1.0^{bc}	25.4±0.8^{abc}	2219±76^{b}	5446±93^{bc}
	**P1**	57.8±0.6^{ab}	25.6±0.5^{ab}	2255±23^{b}	5584±54^{ab}
	**P2**	58.5±1.0^{ab}	25.7±0.7^{ab}	2286±91^{b}	5655±97^{ab}

Within each column, the data followed with different letters are statistically different at the 0.05 probability level.

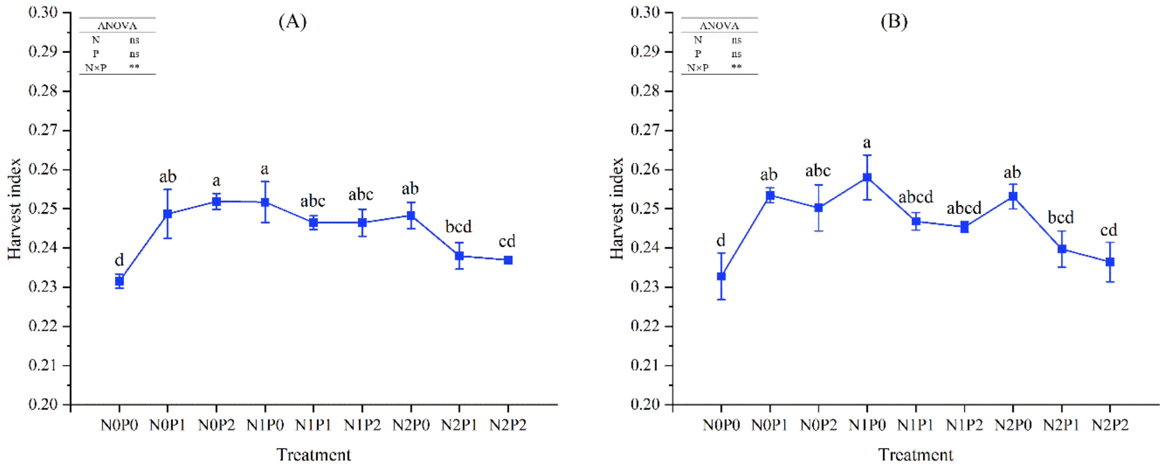

Figure S1. Effects of nitrogen and phosphorus on harvest index of sorghum grown in saline soils in 2021 and 2023. (A) 2021; (B) 2023; ns: non-significant difference; **: significant difference at $P \leq 0.01$.

Table S3. Effects of nitrogen and phosphorus on SPAD reading and NSC of sorghum at three growing stages grown in saline soils in 2021 and 2023

Nitrogen	Phosphorus	SPAD reading			Aerial NSC content (mg g^{-1})		
		Seedling	Jointing	Maturity	Seedling	Jointing	Maturity
**2021**							
**N0**	**P0**	30.0±0.6^{f}	37.3±0.6^{e}	28.0±0.3^{f}	167.9±1.2^{f}	77.0±0.9^{e}	268.8±4.4^{e}
	**P1**	32.6±0.4^{de}	41.7±0.9^{bc}	30.0±0.2^{def}	199.0±2.2^{d}	87.7±1.7^{d}	332.0±10.1^{c}
	**P2**	31.4±0.8^{ef}	38.9±0.8^{de}	29.2±1.2^{ef}	201.4±4.0^{d}	93.3±1.1^{c}	315.3±6.4^{cd}
**N1**	**P0**	33.2±0.6^{de}	40.1±0.5^{cd}	30.2±1.2^{def}	188.1±1.2^{e}	88.4±0.9^{d}	309.8±5.0^{d}
	**P1**	37.2±0.6^{ab}	45.7±0.8^{a}	34.4±0.9^{ab}	229.4±2.7^{ab}	105.3±1.4^{a}	375.9±3.7^{a}
	**P2**	35.4±0.5^{bc}	43.1±0.7^{b}	32.4±0.8^{bcd}	216.1±1.8^{c}	98.7±0.6^{b}	351.2±2.9^{b}
**N2**	**P0**	33.7±0.5^{cd}	42.2±0.9^{bc}	31.1±0.8^{cde}	190.1±2.5^{e}	88.7±1.6^{d}	325.1±4.7^{cd}
	**P1**	37.6±0.7^{a}	45.9±1.0^{a}	33.3±0.4^{bc}	225.3±4.1^{b}	99.3±1.1^{b}	356.6±7.7^{b}
	**P2**	38.9±0.7^{a}	47.4±0.3^{a}	36.1±0.8^{a}	235.1±1.8^{a}	106.6±0.7^{a}	385.5±9.1^{a}
**2023**							
**N0**	**P0**	30.5±0.3^{g}	37.1±0.4^{e}	28.4±0.7^{f}	163.3±1.8^{g}	78.1±1.6^{e}	261.3±7.3^{e}
	**P1**	32.5±0.4^{f}	41.1±0.1^{c}	30.7±0.5^{de}	192.8±0.7^{de}	85.6±1.6^{d}	318.9±8.1^{c}
	**P2**	32.1±0.4^{f}	38.6±0.7^{de}	30.0±0.1^{e}	200.3±4.4^{cd}	93.0±0.5^{c}	301.3±6.5^{d}
**N1**	**P0**	33.7±0.2^{e}	39.4±0.2^{d}	30.8±0.3^{de}	181.7±1.9^{f}	87.1±1.7^{d}	294.4±2.9^{d}
	**P1**	37.2±0.5^{bc}	45.5±0.3^{a}	34.1±0.6^{b}	223.9±1.1^{a}	102.5±2.0^{ab}	356.9±7.6^{a}
	**P2**	36.7±0.3^{c}	43.2±0.6^{b}	32.3±0.4^{c}	206.1±2.8^{c}	97.4±2.2^{bc}	337.9±3.3^{b}
**N2**	**P0**	35.0±0.6^{d}	42.4±0.9^{bc}	31.7±0.3^{cd}	190.0±1.7^{e}	86.7±2.0^{d}	310.8±4.5^{cd}
	**P1**	38.4±0.5^{ab}	46.3±0.3^{a}	32.9±0.4^{c}	215.9±2.5^{b}	98.4±1.9^{bc}	339.9±2.8^{b}
	**P2**	39.3±0.2^{a}	46.8±0.5^{a}	36.7±0.4^{a}	227.1±3.8^{a}	106.5±2.8^{a}	363.2±1.4^{a}

Within each sampling date, the data followed with different letters are statistically different at the 0.05 probability level.

Table S4. Effects of nitrogen and phosphorus on aerial N accumulation and NUE of sorghum at three growing stages grown in saline soils in 2021 and 2023

Nitrogen	Phosphorus	Aerial N accumulation (kg ha^{-1})			NUE
		Seedling	Jointing	Maturity	(kg kg^{-1})
**2021**					
**N0**	**P0**	1.989±0.044^{e}	11.59±0.30^{g}	126.5±0.4^{f}	35.9±0.4^{a}
	**P1**	2.320±0.036^{d}	13.61±0.31^{e}	151.2±1.8^{e}	36.8±0.7^{a}
	**P2**	2.211±0.026^{d}	12.599±0.17^{f}	145.4±4.1^{e}	36.8±0.2^{a}
**N1**	**P0**	2.379±0.075^{cd}	14.04±0.26^{de}	153.0±1.1^{de}	35.4±0.7^{a}
	**P1**	3.089±0.056^{a}	17.08±0.32^{b}	185.7±2.1^{ab}	32.1±0.7^{cd}
	**P2**	2.806±0.038^{b}	15.99±0.25^{c}	174.0±3.8^{c}	33.3±0.5^{bc}
**N2**	**P0**	2.504±0.084^{c}	14.51±0.13^{d}	160.5±2.3^{d}	33.9±0.4^{b}
	**P1**	3.102±0.066^{a}	17.11±0.15^{b}	181.9±3.1^{b}	31.0±0.2^{de}
	**P2**	3.202±0.033^{a}	18.50±0.35^{a}	190.9±2.8^{a}	30.1±0.2^{e}
**2023**					
**N0**	**P0**	1.876±0.056^{d}	11.16±0.31^{e}	120.1±0.5^{g}	37.6±0.5^{ab}
	**P1**	2.147±0.030^{c}	12.93±0.21^{d}	142.5±1.9^{e}	39.2±0.8^{a}
	**P2**	2.055±0.027^{cd}	11.87±0.43^{e}	136.0±2.7^{f}	38.3±0.5^{a}
**N1**	**P0**	2.204±0.059^{c}	13.18±0.23^{d}	144.0±2.3^{de}	37.9±0.7^{ab}
	**P1**	2.843±0.080^{a}	16.17±0.30^{b}	174.2±1.9^{ab}	33.7±0.3^{de}
	**P2**	2.518±0.081^{b}	14.96±0.24^{c}	159.7±3.0^{c}	35.3±0.4^{cd}
**N2**	**P0**	2.244±0.044^{c}	13.50±0.32^{d}	149.0±1.3^{d}	36.5±0.4^{bc}
	**P1**	2.888±0.054^{a}	16.26±0.26^{b}	170.2±2.6^{b}	32.8±0.6^{ef}
	**P2**	2.967±0.088^{a}	17.42±0.36^{a}	178.9±0.8^{a}	31.6±0.6^{f}

NUE: nitrogen use efficiency. Within each sampling date, the data followed with different letters are statistically different at the 0.05 probability level.

Table S5. Effects of nitrogen and phosphorus on aerial P accumulation and PUE of sorghum at three growing stages grown in saline soils in 2021 and 2023

Nitrogen	Phosphorus	Aerial P accumulation (kg ha^{-1})			PUE
		Seedling	Jointing	Maturity	(kg kg^{-1})
**2021**					
**N0**	**P0**	0.312±0.004^{e}	1.984±0.070^{e}	34.3±0.4^{e}	132.5±0.7^{c}
	**P1**	0.394±0.008^{c}	2.353±0.038^{c}	41.6±0.8^{c}	134.0±3.6^{bc}
	**P2**	0.375±0.004^{cd}	2.220±0.012^{d}	40.1±0.7^{cd}	133.1±1.4^{bc}
**N1**	**P0**	0.354±0.011^{d}	2.195±0.008^{d}	38.4±0.9^{d}	140.8±1.0^{a}
	**P1**	0.489±0.014^{a}	2.748±0.031^{b}	47.5±0.5^{ab}	125.5±1.2^{d}
	**P2**	0.485±0.006^{a}	2.725±0.006^{b}	48.3±0.7^{a}	119.9±2.0^{de}
**N2**	**P0**	0.347±0.004^{d}	2.209±0.027^{d}	39.2±0.7^{d}	138.8±1.6^{ab}
	**P1**	0.454±0.012^{b}	2.685±0.054^{b}	46.0±0.7^{b}	122.6±0.4^{de}
	**P2**	0.501±0.012^{a}	2.881±0.058^{a}	48.9±0.7^{a}	117.9±2.5^{e}
**2023**					
**N0**	**P0**	0.290±0.005^{e}	1.862±0.008^{e}	31.2±0.5^{e}	145.0±3.6^{cd}
	**P1**	0.358±0.008^{c}	2.164±0.036^{c}	37.3±0.6^{c}	150.0±3.8^{abc}
	**P2**	0.338±0.006^{cd}	2.039±0.054^{d}	35.6±1.4^{cd}	146.6±2.1^{bcd}
**N1**	**P0**	0.322±0.009^{d}	2.021±0.011^{d}	34.7±0.5^{d}	157.0±2.0^{a}
	**P1**	0.450±0.008^{a}	2.556±0.031^{b}	42.4±0.1^{ab}	138.4±1.2^{de}
	**P2**	0.442±0.010^{a}	2.511±0.043^{b}	43.2±0.4^{ab}	130.3±1.1^{ef}
**N2**	**P0**	0.315±0.004^{d}	2.031±0.027^{d}	35.3±1.0^{cd}	154.5±2.8^{ab}
	**P1**	0.418±0.003^{b}	2.501±0.035^{b}	41.2±0.7^{b}	135.7±3.3^{ef}
	**P2**	0.460±0.013^{a}	2.677±0.029^{a}	43.8±0.7^{a}	129.3±3.5^{f}

PUE: phosphorus use efficiency. Within each sampling date, the data followed with different letters are statistically different at the 0.05 probability level.

Table S6. Effects of nitrogen and phosphorus on aerial K accumulation and KUE of sorghum at three growing stages grown in saline soils in 2021 and 2023

Nitrogen	Phosphorus	Aerial K accumulation (kg ha^{-1})			KUE
		Seedling	Jointing	Maturity	(kg kg^{-1})
2021					
N0	P0	1.661±0.011^{d}	7.78±0.16^{e}	104.3±0.9^{f}	43.6±0.2^{ab}
	P1	2.167±0.048^{ab}	9.82±0.22^{ab}	131.7±0.9^{ab}	42.3±1.0^{b}
	P2	1.881±0.047^{c}	8.67±0.21^{d}	119.6±1.2^{e}	44.7±0.7^{a}
N1	P0	2.021±0.080^{abc}	9.36±0.24^{bc}	124.1±1.1^{d}	43.6±0.6^{ab}
	P1	2.206±0.051^{a}	9.93±0.09^{a}	134.7±0.8^{a}	44.3±0.7^{ab}
	P2	2.034±0.040^{abc}	9.65±0.14^{ab}	129.3±0.9^{bc}	44.8±1.1^{a}
N2	P0	2.004±0.028^{bc}	9.10±0.17^{cd}	125.0±3.1^{cd}	43.5±0.2^{ab}
	P1	2.163±0.064^{ab}	9.80±0.12^{ab}	129.1±1.7^{bc}	43.7±0.7^{ab}
	P2	2.166±0.091^{ab}	9.93±0.11^{a}	130.9±0.4^{ab}	44.0±0.4^{ab}
2023					
N0	P0	1.509±0.036^{d}	7.38±0.06^{e}	96.5±0.7^{e}	46.8±1.0^{a}
	P1	1.927±0.020^{ab}	9.22±0.14^{ab}	119.5±2.4^{a}	46.7±0.4^{a}
	P2	1.678±0.036^{c}	8.11±0.15^{d}	108.8±1.4^{d}	48.0±2.0^{a}
N1	P0	1.798±0.052^{bc}	8.72±0.07^{bc}	112.3±2.3^{cd}	48.5±0.7^{a}
	P1	1.961±0.059^{a}	9.29±0.36^{a}	122.8±1.1^{a}	47.8±0.6^{a}
	P2	1.810±0.068^{abc}	8.98±0.04^{ab}	117.6±1.5^{abc}	47.8±0.4^{a}
N2	P0	1.766±0.036^{c}	8.38±0.09^{cd}	113.7±1.3^{bcd}	47.9±0.9^{a}
	P1	1.932±0.020^{ab}	9.16±0.14^{ab}	117.9±2.1^{ab}	47.4±1.3^{a}
	P2	1.937±0.063^{ab}	9.27±0.21^{ab}	119.9±2.3^{a}	47.2±1.1^{a}

KUE: potassium use efficiency. Within each sampling date, the data followed with different letters are statistically different at the 0.05 probability level.

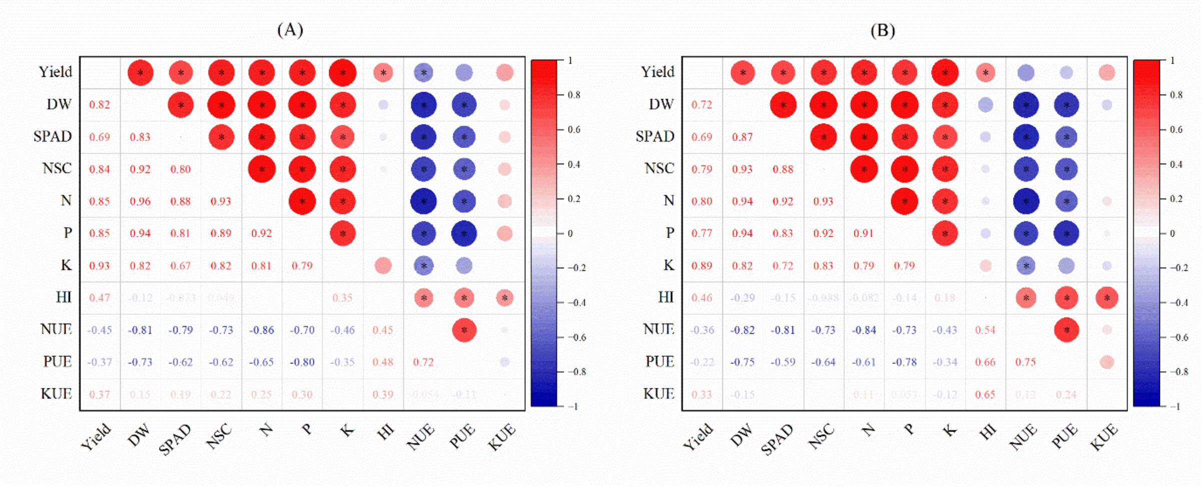

Figure S2. Relationships between all the measured characteristics of sorghum grown in saline soils in 2021 and 2023. (A) 2021; (B) 2023; DW: Dry weight; N: Aerial N accumulation; P: Aerial P accumulation; K: Aerial K accumulation. *: significant difference at $P \leq 0.05$.
